# Supplementary material for: Iterative improvement in the automatic modular design of robot swarms
Source: PeerJ Comput Sci. 2020 Dec 7;6:e322. doi: 10.7717/peerj-cs.322 (PMC7924708; doi:10.7717/peerj-cs.322)
Supplement: Supplemental Information 3 [file peerj-cs-06-322-s003.zip › argos3/doc/api/standalone/a00432_source.html]

ARGoS: plugins/robots/eye-bot/simulator/qtopengl\_eyebot.cpp Source File


- Main Page
- Related Pages
- Namespaces
- Classes
- Files

- File List
- File Members

# plugins/robots/eye-bot/simulator/qtopengl\_eyebot.cpp

Go to the documentation of this file.

```
00001 
00007 #include "qtopengl_eyebot.h"
00008 #include "eyebot_entity.h"
00009 #include <argos3/core/simulator/entity/embodied_entity.h>
00010 #include <argos3/core/utility/math/vector2.h>
00011 #include <argos3/core/utility/math/vector3.h>
00012 #include <argos3/plugins/simulator/entities/led_equipped_entity.h>
00013 #include <argos3/plugins/simulator/visualizations/qt-opengl/qtopengl_widget.h>
00014 
00015 namespace argos {
00016 
00017    /****************************************/
00018    /****************************************/
00019 
00020    /* All measures are in meters */
00021 
00022    /* Legs */
00023    static const Real LEG_HEIGHT         = 0.166f;
00024    static const Real LEG_WIDTH          = 0.02f;
00025    static const Real LEG_HALF_WIDTH     = LEG_WIDTH * 0.5f;
00026    static const Real LEG_DEPTH          = 0.005f;
00027    static const Real LEG_HALF_DEPTH     = LEG_DEPTH * 0.5f;
00028 
00029    /* Ring */
00030    static const Real RING_OUTER_RADIUS  = 0.25f;
00031    static const Real RING_INNER_RADIUS  = 0.237f;
00032    static const Real RING_HEIGHT        = 0.015f;
00033    static const Real RING_ELEVATION     = LEG_HEIGHT - RING_HEIGHT;
00034 
00035    /* Led */
00036    static const Real LED_HEIGHT               = 0.01f;
00037    static const Real LED_SIDE                 = RING_HEIGHT;
00038    static const Real LED_HALF_SIDE            = LED_SIDE * 0.5f;
00039    static const Real LED_SIDE_RING_ELEVATION = RING_ELEVATION + LED_HALF_SIDE;
00040    static const Real LED_BOTTOM_RING_ELEVATION = RING_ELEVATION - LED_HEIGHT;
00041    static const Real LED_BOTTOM_RING_DISTANCE  = RING_OUTER_RADIUS - LEG_HALF_WIDTH;
00042 
00043    /* Main rod */
00044    static const Real ROD_SIDE           = 0.07f;
00045    static const Real ROD_HALF_SIDE      = ROD_SIDE * 0.5f;
00046    static const Real ROD_HEIGHT         = 0.4f;
00047    static const Real ROD_ELEVATION      = LEG_HEIGHT;
00048 
00049    /****************************************/
00050    /****************************************/
00051 
00052    CQTOpenGLEyeBot::CQTOpenGLEyeBot() :
00053       m_unVertices(40),
00054       m_fLEDAngleSlice(360.0f / 16.0f) {
00055       /* Reserve the needed display lists */
00056       m_unLists = glGenLists(4);
00057 
00058       /* Assign indices for better referencing (later) */
00059       m_unLegList      = m_unLists;
00060       m_unBodyList     = m_unLists + 1;
00061       m_unSideLEDList = m_unLists + 2;
00062       m_unBottomLEDList = m_unLists + 3;
00063 
00064       /* Create the led display list */
00065       glNewList(m_unLegList, GL_COMPILE);
00066       MakeLeg();
00067       glEndList();
00068 
00069       /* Create the body display list */
00070       glNewList(m_unBodyList, GL_COMPILE);
00071       DrawBody();
00072       glEndList();
00073 
00074       /* Create the side LED display list */
00075       glNewList(m_unSideLEDList, GL_COMPILE);
00076       DrawSideLED();
00077       glEndList();
00078 
00079       /* Create the bottom LED display list */
00080       glNewList(m_unBottomLEDList, GL_COMPILE);
00081       DrawBottomLED();
00082       glEndList();
00083    }
00084 
00085    /****************************************/
00086    /****************************************/
00087 
00088    CQTOpenGLEyeBot::~CQTOpenGLEyeBot() {
00089       glDeleteLists(m_unLists, 4);
00090    }
00091 
00092    /****************************************/
00093    /****************************************/
00094 
00095    void CQTOpenGLEyeBot::Draw(CEyeBotEntity& c_entity) {
00096       SetBodyMaterial();
00097       /* Place the legs */
00098       glPushMatrix();
00099       glRotatef(45.0f, 0.0f, 0.0f, 1.0f);
00100       glTranslatef(RING_OUTER_RADIUS, 0.0f, 0.0f);
00101       glCallList(m_unLegList);
00102       glPopMatrix();
00103       glPushMatrix();
00104       glRotatef(135.0f, 0.0f, 0.0f, 1.0f);
00105       glTranslatef(RING_OUTER_RADIUS, 0.0f, 0.0f);
00106       glCallList(m_unLegList);
00107       glPopMatrix();
00108       glPushMatrix();
00109       glRotatef(225.0f, 0.0f, 0.0f, 1.0f);
00110       glTranslatef(RING_OUTER_RADIUS, 0.0f, 0.0f);
00111       glCallList(m_unLegList);
00112       glPopMatrix();
00113       glPushMatrix();
00114       glRotatef(315.0f, 0.0f, 0.0f, 1.0f);
00115       glTranslatef(RING_OUTER_RADIUS, 0.0f, 0.0f);
00116       glCallList(m_unLegList);
00117       glPopMatrix();
00118       /* Place the body */
00119       glCallList(m_unBodyList);
00120       /* Place the LEDs */
00121       glPushMatrix();
00122       CLEDEquippedEntity& cLEDEquippedEntity = c_entity.GetLEDEquippedEntity();
00123       glRotatef(m_fLEDAngleSlice * 0.5f, 0.0f, 0.0f, 1.0f);
00124       const CColor& cUpColor = cLEDEquippedEntity.GetLED(0).GetColor();
00125       const CColor& cLowColor = cLEDEquippedEntity.GetLED(16).GetColor();
00126       SetLEDMaterial(cUpColor.GetRed(),
00127                      cUpColor.GetGreen(),
00128                      cUpColor.GetBlue());
00129       glCallList(m_unSideLEDList);
00130       SetLEDMaterial(cLowColor.GetRed(),
00131                      cLowColor.GetGreen(),
00132                      cLowColor.GetBlue());
00133       glCallList(m_unBottomLEDList);
00134       for(UInt32 i = 1; i < 16; i++) {
00135          glRotatef(m_fLEDAngleSlice, 0.0f, 0.0f, 1.0f);
00136          const CColor& cUpColor = cLEDEquippedEntity.GetLED(i).GetColor();
00137          const CColor& cLowColor = cLEDEquippedEntity.GetLED(i+16).GetColor();
00138          SetLEDMaterial(cUpColor.GetRed(),
00139                         cUpColor.GetGreen(),
00140                         cUpColor.GetBlue());
00141          glCallList(m_unSideLEDList);
00142          SetLEDMaterial(cLowColor.GetRed(),
00143                         cLowColor.GetGreen(),
00144                         cLowColor.GetBlue());
00145          glCallList(m_unBottomLEDList);
00146       }
00147       glRotatef(135.0f, 0.0f, 0.0f, 1.0f);
00148       glTranslatef(-0.3*RING_OUTER_RADIUS, 0.0f, 0.0f);
00149       const CColor& cBottomLedColor = cLEDEquippedEntity.GetLED(32).GetColor();
00150       SetLEDMaterial(cBottomLedColor.GetRed(),
00151                      cBottomLedColor.GetGreen(),
00152                      cBottomLedColor.GetBlue());
00153       glCallList(m_unBottomLEDList);
00154       glPopMatrix();
00155    }
00156 
00157    /****************************************/
00158    /****************************************/
00159 
00160    void CQTOpenGLEyeBot::SetLEDMaterial(GLfloat f_red, GLfloat f_green, GLfloat f_blue) {
00161       const GLfloat fEmissionFactor = 10.0f;
00162       const GLfloat pfColor[]     = {                    f_red,                   f_green,                   f_blue, 1.0f };
00163       const GLfloat pfSpecular[]  = {                     0.0f,                      0.0f,                     0.0f, 1.0f };
00164       const GLfloat pfShininess[] = {                     0.0f                                                            };
00165       const GLfloat pfEmission[]  = {  f_red * fEmissionFactor, f_green * fEmissionFactor, f_blue * fEmissionFactor, 1.0f };
00166       glMaterialfv(GL_FRONT_AND_BACK, GL_AMBIENT_AND_DIFFUSE, pfColor);
00167       glMaterialfv(GL_FRONT_AND_BACK, GL_SPECULAR,            pfSpecular);
00168       glMaterialfv(GL_FRONT_AND_BACK, GL_SHININESS,           pfShininess);
00169       glMaterialfv(GL_FRONT_AND_BACK, GL_EMISSION,            pfEmission);
00170    }
00171 
00172    /****************************************/
00173    /****************************************/
00174 
00175    void CQTOpenGLEyeBot::SetBodyMaterial() {
00176       const GLfloat pfColor[]     = {   0.25f, 0.25f, 0.25f, 1.0f };
00177       const GLfloat pfSpecular[]  = {   0.9f,  0.9f,  0.9f,  1.0f };
00178       const GLfloat pfShininess[] = { 100.0f                   };
00179       const GLfloat pfEmission[]  = {   0.0f, 0.0f, 0.0f, 1.0f };
00180       glMaterialfv(GL_FRONT_AND_BACK, GL_AMBIENT_AND_DIFFUSE, pfColor);
00181       glMaterialfv(GL_FRONT_AND_BACK, GL_SPECULAR,            pfSpecular);
00182       glMaterialfv(GL_FRONT_AND_BACK, GL_SHININESS,           pfShininess);
00183       glMaterialfv(GL_FRONT_AND_BACK, GL_EMISSION,            pfEmission);
00184    }
00185 
00186    /****************************************/
00187    /****************************************/
00188 
00189    void CQTOpenGLEyeBot::MakeLeg() {
00190       /* Top face */
00191       glBegin(GL_QUADS);
00192       glNormal3f(0.0f, 0.0f, 1.0f);
00193       glVertex3f(-LEG_HALF_WIDTH, -LEG_HALF_DEPTH, LEG_HEIGHT);
00194       glVertex3f( LEG_HALF_WIDTH, -LEG_HALF_DEPTH, LEG_HEIGHT);
00195       glVertex3f( LEG_HALF_WIDTH,  LEG_HALF_DEPTH, LEG_HEIGHT);
00196       glVertex3f(-LEG_HALF_WIDTH,  LEG_HALF_DEPTH, LEG_HEIGHT);
00197       glEnd();
00198       /* Bottom face */
00199       glBegin(GL_QUADS);
00200       glNormal3f(0.0f, 0.0f, -1.0f);
00201       glVertex3f(-LEG_HALF_WIDTH, -LEG_HALF_DEPTH, 0.0f);
00202       glVertex3f(-LEG_HALF_WIDTH,  LEG_HALF_DEPTH, 0.0f);
00203       glVertex3f( LEG_HALF_WIDTH,  LEG_HALF_DEPTH, 0.0f);
00204       glVertex3f( LEG_HALF_WIDTH, -LEG_HALF_DEPTH, 0.0f);
00205       glEnd();
00206       /* This part covers the faces (South, East, North, West) */
00207       glBegin(GL_QUAD_STRIP);
00208       /* Starting vertex */
00209       glNormal3f(0.0f, -1.0f, 0.0f);
00210       glVertex3f(-LEG_HALF_WIDTH, -LEG_HALF_DEPTH, LEG_HEIGHT);
00211       glVertex3f(-LEG_HALF_WIDTH, -LEG_HALF_DEPTH, 0.0f);
00212       /* South face */
00213       glVertex3f( LEG_HALF_WIDTH, -LEG_HALF_DEPTH, LEG_HEIGHT);
00214       glVertex3f( LEG_HALF_WIDTH, -LEG_HALF_DEPTH, 0.0f);
00215       /* East face */
00216       glNormal3f(1.0f, 0.0f, 0.0f);
00217       glVertex3f( LEG_HALF_WIDTH,  LEG_HALF_DEPTH, LEG_HEIGHT);
00218       glVertex3f( LEG_HALF_WIDTH,  LEG_HALF_DEPTH, 0.0f);
00219       /* North face */
00220       glNormal3f(0.0f, 1.0f, 0.0f);
00221       glVertex3f(-LEG_HALF_WIDTH,  LEG_HALF_DEPTH, LEG_HEIGHT);
00222       glVertex3f(-LEG_HALF_WIDTH,  LEG_HALF_DEPTH, 0.0f);
00223       /* West face */
00224       glNormal3f(-1.0f, 0.0f, 0.0f);
00225       glVertex3f(-LEG_HALF_WIDTH, -LEG_HALF_DEPTH, LEG_HEIGHT);
00226       glVertex3f(-LEG_HALF_WIDTH, -LEG_HALF_DEPTH, 0.0f);
00227       glEnd();
00228    }
00229 
00230    /****************************************/
00231    /****************************************/
00232 
00233    void CQTOpenGLEyeBot::DrawSideLED() {
00234       /* Draw it as a tiny pyramid pointing to X
00235          (with no base, cause it's not visible anyway) */
00236       glBegin(GL_TRIANGLES);
00237       /* Top */
00238       glVertex3f(LED_HEIGHT + RING_OUTER_RADIUS,           0.0f, LED_SIDE_RING_ELEVATION                );
00239       glVertex3f(             RING_OUTER_RADIUS,  LED_HALF_SIDE, LED_SIDE_RING_ELEVATION + LED_HALF_SIDE);
00240       glVertex3f(             RING_OUTER_RADIUS, -LED_HALF_SIDE, LED_SIDE_RING_ELEVATION + LED_HALF_SIDE);
00241       /* Bottom */
00242       glVertex3f(LED_HEIGHT + RING_OUTER_RADIUS,           0.0f, LED_SIDE_RING_ELEVATION                );
00243       glVertex3f(             RING_OUTER_RADIUS, -LED_HALF_SIDE, LED_SIDE_RING_ELEVATION - LED_HALF_SIDE);
00244       glVertex3f(             RING_OUTER_RADIUS,  LED_HALF_SIDE, LED_SIDE_RING_ELEVATION - LED_HALF_SIDE);
00245       /* Left */
00246       glVertex3f(LED_HEIGHT + RING_OUTER_RADIUS,           0.0f, LED_SIDE_RING_ELEVATION                );
00247       glVertex3f(             RING_OUTER_RADIUS,  LED_HALF_SIDE, LED_SIDE_RING_ELEVATION - LED_HALF_SIDE);
00248       glVertex3f(             RING_OUTER_RADIUS,  LED_HALF_SIDE, LED_SIDE_RING_ELEVATION + LED_HALF_SIDE);
00249       /* Right */
00250       glVertex3f(LED_HEIGHT + RING_OUTER_RADIUS,           0.0f, LED_SIDE_RING_ELEVATION                );
00251       glVertex3f(             RING_OUTER_RADIUS, -LED_HALF_SIDE, LED_SIDE_RING_ELEVATION + LED_HALF_SIDE);
00252       glVertex3f(             RING_OUTER_RADIUS, -LED_HALF_SIDE, LED_SIDE_RING_ELEVATION - LED_HALF_SIDE);
00253       glEnd();
00254    }
00255 
00256    /****************************************/
00257    /****************************************/
00258 
00259    void CQTOpenGLEyeBot::DrawBottomLED() {
00260       /* Draw it as a tiny pyramid pointing to Z downwards
00261          (with no base, cause it's not visible anyway) */
00262       glBegin(GL_TRIANGLES);
00263       /* North */
00264       glVertex3f(LED_BOTTOM_RING_DISTANCE, 0.0f, LED_BOTTOM_RING_ELEVATION);
00265       glVertex3f(RING_OUTER_RADIUS,  LED_HALF_SIDE, RING_ELEVATION);
00266       glVertex3f(RING_OUTER_RADIUS, -LED_HALF_SIDE, RING_ELEVATION);
00267       /* South */
00268       glVertex3f(LED_BOTTOM_RING_DISTANCE, 0.0f, LED_BOTTOM_RING_ELEVATION);
00269       glVertex3f(RING_OUTER_RADIUS - LED_SIDE, -LED_HALF_SIDE, RING_ELEVATION);
00270       glVertex3f(RING_OUTER_RADIUS - LED_SIDE,  LED_HALF_SIDE, RING_ELEVATION);
00271       /* West */
00272       glVertex3f(LED_BOTTOM_RING_DISTANCE, 0.0f, LED_BOTTOM_RING_ELEVATION);
00273       glVertex3f(RING_OUTER_RADIUS - LED_SIDE, LED_HALF_SIDE, RING_ELEVATION);
00274       glVertex3f(RING_OUTER_RADIUS           , LED_HALF_SIDE, RING_ELEVATION);
00275       /* East */
00276       glVertex3f(LED_BOTTOM_RING_DISTANCE, 0.0f, LED_BOTTOM_RING_ELEVATION);
00277       glVertex3f(RING_OUTER_RADIUS           , -LED_HALF_SIDE, RING_ELEVATION);
00278       glVertex3f(RING_OUTER_RADIUS - LED_SIDE, -LED_HALF_SIDE, RING_ELEVATION);
00279       glEnd();
00280    }
00281 
00282    /****************************************/
00283    /****************************************/
00284 
00285    void CQTOpenGLEyeBot::DrawBody() {
00286       /* Draw the ring */
00287       CVector2 cVertex(RING_OUTER_RADIUS, 0.0f);
00288       CRadians cAngle(-CRadians::TWO_PI / m_unVertices);
00289       /* Bottom part */
00290       glBegin(GL_POLYGON);
00291       glNormal3f(0.0f, 0.0f, -1.0f);
00292       for(GLuint i = 0; i <= m_unVertices; i++) {
00293          glVertex3f(cVertex.GetX(), cVertex.GetY(), RING_ELEVATION);
00294          cVertex.Rotate(cAngle);
00295       }
00296       glEnd();
00297       /* Side surface */
00298       cAngle = -cAngle;
00299       CVector2 cNormal(1.0f, 0.0f);
00300       cVertex.Set(RING_OUTER_RADIUS, 0.0f);
00301       glBegin(GL_QUAD_STRIP);
00302       for(GLuint i = 0; i <= m_unVertices; i++) {
00303          glNormal3f(cNormal.GetX(), cNormal.GetY(), 0.0f);
00304          glVertex3f(cVertex.GetX(), cVertex.GetY(), RING_ELEVATION + RING_HEIGHT);
00305          glVertex3f(cVertex.GetX(), cVertex.GetY(), RING_ELEVATION);
00306          cVertex.Rotate(cAngle);
00307          cNormal.Rotate(cAngle);
00308       }
00309       glEnd();
00310       /* Top part */
00311       glBegin(GL_POLYGON);
00312       glNormal3f(0.0f, 0.0f, 1.0f);
00313       cVertex.Set(RING_OUTER_RADIUS, 0.0f);
00314       for(GLuint i = 0; i <= m_unVertices; i++) {
00315          glVertex3f(cVertex.GetX(), cVertex.GetY(), RING_ELEVATION + RING_HEIGHT);
00316          cVertex.Rotate(cAngle);
00317       }
00318       glEnd();
00319       /* Draw the rod */
00320       /* Top face */
00321       glBegin(GL_QUADS);
00322       glNormal3f(0.0f, 0.0f, 1.0f);
00323       glVertex3f(-ROD_HALF_SIDE, -ROD_HALF_SIDE, ROD_ELEVATION+ROD_HEIGHT);
00324       glVertex3f( ROD_HALF_SIDE, -ROD_HALF_SIDE, ROD_ELEVATION+ROD_HEIGHT);
00325       glVertex3f( ROD_HALF_SIDE,  ROD_HALF_SIDE, ROD_ELEVATION+ROD_HEIGHT);
00326       glVertex3f(-ROD_HALF_SIDE,  ROD_HALF_SIDE, ROD_ELEVATION+ROD_HEIGHT);
00327       glEnd();
00328       /* This part covers the faces (South, East, North, West) */
00329       glBegin(GL_QUAD_STRIP);
00330       /* Starting vertex */
00331       glNormal3f(0.0f, -1.0f, 0.0f);
00332       glVertex3f(-ROD_HALF_SIDE, -ROD_HALF_SIDE, ROD_ELEVATION+ROD_HEIGHT);
00333       glVertex3f(-ROD_HALF_SIDE, -ROD_HALF_SIDE, ROD_ELEVATION);
00334       /* South face */
00335       glVertex3f( ROD_HALF_SIDE, -ROD_HALF_SIDE, ROD_ELEVATION+ROD_HEIGHT);
00336       glVertex3f( ROD_HALF_SIDE, -ROD_HALF_SIDE, ROD_ELEVATION);
00337       /* East face */
00338       glNormal3f(1.0f, 0.0f, 0.0f);
00339       glVertex3f( ROD_HALF_SIDE,  ROD_HALF_SIDE, ROD_ELEVATION+ROD_HEIGHT);
00340       glVertex3f( ROD_HALF_SIDE,  ROD_HALF_SIDE, ROD_ELEVATION);
00341       /* North face */
00342       glNormal3f(0.0f, 1.0f, 0.0f);
00343       glVertex3f(-ROD_HALF_SIDE,  ROD_HALF_SIDE, ROD_ELEVATION+ROD_HEIGHT);
00344       glVertex3f(-ROD_HALF_SIDE,  ROD_HALF_SIDE, ROD_ELEVATION);
00345       /* West face */
00346       glNormal3f(-1.0f, 0.0f, 0.0f);
00347       glVertex3f(-ROD_HALF_SIDE, -ROD_HALF_SIDE, ROD_ELEVATION+ROD_HEIGHT);
00348       glVertex3f(-ROD_HALF_SIDE, -ROD_HALF_SIDE, ROD_ELEVATION);
00349       glEnd();
00350    }
00351 
00352    /****************************************/
00353    /****************************************/
00354 
00355    class CQTOpenGLOperationDrawEyeBotNormal : public CQTOpenGLOperationDrawNormal {
00356    public:
00357       void ApplyTo(CQTOpenGLWidget& c_visualization,
00358                    CEyeBotEntity& c_entity) {
00359          static CQTOpenGLEyeBot m_cModel;
00360          c_visualization.DrawRays(c_entity.GetControllableEntity());
00361          c_visualization.DrawEntity(c_entity.GetEmbodiedEntity());
00362          m_cModel.Draw(c_entity);
00363       }
00364    };
00365 
00366    class CQTOpenGLOperationDrawEyeBotSelected : public CQTOpenGLOperationDrawSelected {
00367    public:
00368       void ApplyTo(CQTOpenGLWidget& c_visualization,
00369                    CEyeBotEntity& c_entity) {
00370          c_visualization.DrawBoundingBox(c_entity.GetEmbodiedEntity());
00371       }
00372    };
00373 
00374    REGISTER_QTOPENGL_ENTITY_OPERATION(CQTOpenGLOperationDrawNormal, CQTOpenGLOperationDrawEyeBotNormal, CEyeBotEntity);
00375 
00376    REGISTER_QTOPENGL_ENTITY_OPERATION(CQTOpenGLOperationDrawSelected, CQTOpenGLOperationDrawEyeBotSelected, CEyeBotEntity);
00377 
00378    /****************************************/
00379    /****************************************/
00380 
00381 }
```

---

Generated on 10 Jul 2018 for ARGoS by 
 1.6.1
